# Editorial: The Psychological and Physiological Benefits of the Arts

**DOI:** 10.3389/fpsyg.2022.840089

**Published:** 2022-03-08

**Authors:** Vicky Karkou, Nisha Sajnani, Hod Orkibi, Jenny M. Groarke, Johanna Czamanski-Cohen, Maria Eugenia Panero, Jennifer Drake, Corinne Jola, Felicity Anne Baker

**Affiliations:** ^1^Research Centre for Arts and Wellbeing, Edge Hill University, Ormskirk, United Kingdom; ^2^Steinhardt School of Culture, Education, and Human Development, New York University, New York, NY, United States; ^3^The School of Creative Arts Therapies, University of Haifa, Haifa, Israel; ^4^School of Psychology, College of Arts, Social Sciences, and Celtic Studies, National University of Ireland Galway, Galway, Ireland; ^5^Office of International Affairs, Yale University, New Haven, CT, United States; ^6^Psychology Department, Brooklyn College, City University of New York, Brooklyn, NY, United States; ^7^Division of Psychology and Forensic Sciences, Abertay University, Dundee, United Kingdom; ^8^Melbourne Conservatorium of Music, The University of Melbourne, Parkville, VIC, Australia

**Keywords:** music, drama, theater, dance, art, creative arts therapies, psychological, physiological

The role of the arts in healing has a long history. From the first cave markings and healing performance rituals, the arts have been used to represent, communicate, and elevate human experience. In recognition of the power of the arts to improve wellbeing, the World Health Organisation (WHO) published a report on the evidence for the arts in improving health and wellbeing (Fancourt and Finn, 2019). Furthermore, the WHO has officially launched their arts and health program, organizing events, supporting research and raising awareness (Arts and Health). It is therefore not accidental that this Research Topic, that has called for new evidence on the psychological and physiological benefits for the arts, includes a commentary by Christopher Bailey, the lead for arts and health at the WHO. This call for evidence also includes studies on the social impact of the arts on families, neighborhoods and communities including research on implicit bias and the role of the arts in reducing social stigma. The result is a portfolio of evidence on both prevention and treatment, for both individuals and groups, in clinical and community-based settings, and across the lifespan.

With an international editorial team of researchers specializing in the psychology of the arts as well as the creative arts therapies, this Research Topic has attracted publications from around the world on different aspects of the arts including visual arts, music, dance and theater alongside studies on the creative arts therapies including music therapy, art therapy, drama therapy, dance movement therapy, and integrated approaches. We maintained this interdisciplinary focus by prioritizing studies that reflect collaborations across countries and between university research partners and community organizations. We are also proud to showcase studies by doctoral students presented jointly with their supervisory teams thus advancing academic and research discourse with direct implications for clinical practice and policy.

In this editorial, studies have been organized by artistic practice with an emphasis on the benefits to individuals and groups in specific settings such as schools, museums, hospitals, age care facilities, and community centers. We also have studies that combine different forms of arts practices; the benefits of these combined interventions are also highlighted in our summary of these studies. This collection of studies affirms the value of the arts in addressing and communicating health concerns and improving quality of life thus further reinforcing the need for national frameworks, such as those developed in the UK (Arts for Health and Wellbeing) and Australia (National Arts and Health Framework), to enhance the profile of the arts and health and promote better integration of arts practice across settings and agencies. Given the range of methods used, this collection also offers important roadmaps for how to measure the impact of the arts on wellbeing and reinforces the need to dedicate funding toward further research in this area.

## Visual Art and Art Therapy

In this Research Topic, the visual arts was the artistic discipline with the greatest number of studies—-with almost a third of the articles on this topic (23 out of 82) focused on either the visual arts, art therapy, or phototherapy, which may be indicative of the proportion of art therapists in the creative arts therapies. While we can only speak to those articles submitted, the sheer volume of articles focused on the visual arts suggests that the visual arts play a crucial role in promoting health, preventing mental and physical illness, and alleviating both acute and chronic conditions.

The articles in this Research Topic focused on a wide variety of populations across the lifespan and included those suffering from psychological, neurological, and physical illnesses as well as healthy children and adults. The studies were conducted in a variety of settings—schools, museums, hospitals, and in the community—suggesting that art can be done anytime and anywhere and included both short-term and long-term interventions. It is important to note that almost half of these articles were conducted with children, families, or art therapists working with children. Perhaps, the focus on young people is not surprising given the pervasiveness of the visual arts in childhood. From a young age children are encouraged to draw, paint, sculpt, and create and it is likely that this is a familiar and comfortable medium for children to express themselves.

The studies covered a range of methodologies from experimental and correlational studies to systematic and comprehensive reviews. A commonality across several studies was the analysis of children's and adult's drawings in terms of what was drawn and how it was drawn. There were also several pieces that focused on participants' experiences.

For the articles that focused on the benefits of engaging in the visual arts, there was a strong emphasis on improving social and emotional wellbeing. For example, engaging in a visual arts intervention was found to reduce neuropsychiatric symptoms and improve quality of life in Alzheimer's patients (Savazzi et al.). A museum-based program focused on drawing faces was shown to improve socio-emotional skills (Kastner et al.); and in separate studies, drawing was shown to help children regulate their emotions (Drake) and promote wellbeing in sailors (Pipere et al.). Thus, emphasizing the potential emotional, cognitive and economic contributions that can come out of bringing the arts back into schools, and introducing them in universities and the workplace, as a means to enhance wellbeing. It may also point to the potential benefit of the social prescribing of the arts, investing in a creative economy and utilizing the arts as preventative measures.

The art therapy and phototherapy articles focused on both the psychological and social benefits of engaging in the arts. Art therapy allowed adolescents in a school setting to express their emotions freely (Harpazi et al.). Art therapy with adults suffering from personality disorders was found to improve their emotional and social functioning (Haeyen et al.), as well as reduce anxiety and depression in adults, when implementing an arts-based dynamic interpersonal therapy (Havsteen-Franklin et al.). Other studies focused on how the development of different art therapy techniques had the potential to improve mental health and wellbeing. For example, the joint drawing technique was demonstrated to aid adults in learning more about their internal representations of closeness and intimacy (Snir et al.). Drawings of hearing families who have a deaf or hard of hearing child were found to assist in better understanding family dynamics (Avrahami-Winaver et al.).

Several additional studies focused on the validation of art therapy techniques that will nonetheless make a contribution to art therapy practice. Kastner et al. developed and validated a tool for the identification, recognition, and expression of emotions associated with traumatic experiences. Wolk et al. interviewed adolescents on their use and perspective of an art therapy technique aimed at improving wellbeing. In a systematic review, Bosgraaf et al. examined art therapists' behavior during sessions finding they respond in eclectic, directive and non-directive ways toward children and adolescents. Finally, in a scoping review, Finkel and Bat-Or found specific core principles of conducting the open studio with a wide variation on its implementation.

It is interesting that the use of digital technologies was found to be on the rise (Zubala et al.) and the incorporation of virtual reality was found to assist in understanding the needs of children and adolescents coping with anxiety and depression (Shamri Zeevi) and was found to be positively received by healthy adults (Kaimal et al.).

Finally, the papers in this special issue span across countries and cover multi-cultural aspects of engaging in the visual arts. In a qualitative examination of perceptions of art therapists working with ultra-orthodox children, significant perceptual and value-based disparities were found between therapists and clients, which pose difficulties and challenges to all participating parties and require therapists to be highly sensitive (Keidar et al.). The incorporation of Chinese art materials in art therapy was deemed to increase self-efficacy and social functioning in Chinese adults with schizophrenia (Tong et al.).

The range and type of impact of the visual arts and art therapy reported in this collection of studies is vast, as is the impact of dance and dance movement therapy.

## Dance and Dance Movement Therapy

Eleven of the published articles dealt with dance and six with dance movement therapy, covering a range of topics, populations and methodologies. It is noteworthy that eight out of 11 studies on dance included the elderly population. For example, Hansen et al. explored the impact of a dance intervention in the elderly, whereas Douse et al., and Shuper-Engelhard conducted intergenerational interventions, highlighting the value of dance in bringing not only families but also communities together.

The value of dance and dance movement therapy for clinical populations is also evident in this call. There are three papers that explore the impact of dance practices for Parkinson's (Fontanesi and DeSouza; Sundstrom and Jola; Bar et al.), five studies on dance movement therapy for adults with mental health problems (Hyvönen et al.; Pylvanainen et al.; Majore-Dusele et al.; Payne and Brook; Kleinlooh et al.) and one study that explores the impact on dance movement therapy with children with Autism Spectrum Disorder (Aithal et al.). The capacity of dance and dance movement therapy to support motor, social, and psychological outcomes for both the general population as well as vulnerable groups is evident in these papers.

Overall, motor outcomes were very wide; from improved gait, balance (Fontanesi and DeSouza), to weight loss and improved fitness levels (Karkou et al.). The latter, for example, showed that a dance intervention used across five different European countries on women recovering from breast cancer positively impacted on weight loss, right and left forearm circumference and hip changes and reduced 6-min walking time, stronger right and left handgrip and further improvements in sit-to-stand and sit-and-reach tests. This, and other studies in this call, suggests that dance is a very successful tool to improve motor control as it engages the whole body and can support physical changes in a pleasurable manner. Whilst the range of motor changes associated with dance can make it challenging to select a limited number of measures, it is noteworthy that motor improvements through dance and dance movement therapy are often achieved alongside improving the quality of life (e.g., Karkou et al.).

Another consistent outcome of dance and dance movement therapy across our Research Topic is related to social components. For example, Shuper-Engelhard assessed the experience in joint dancing between granddaughters and their grandmothers through reflective diaries, video transcriptions, and interview analysis. The study showed that grandmothers' state of mind became more positive and uplifting through dancing together with their granddaughters. Interestingly, the study by Sundstrom and Jola found that playful interaction with young grandchildren in the form of improvisational dance is also an important aspect in the lives of people with Parkinson's. The authors' explored the experiences of people with Parkinson's and their care partners in and beyond dance through qualitative interviews conducted individually with each participant. This novel approach allowed participants to openly discuss their perceptions of dance for Parkinson's programmes, with many expressing doubts on the impact of dance on motor control. Nevertheless, a few participants noted how they could transfer dance movements into their everyday lives. Dancing also presented itself as an opportunity to relate to others through exchanging knowledge and comparing physical abilities, which is particularly relevant considering the many unknowns of the disease.

Dance can certainly support us to turn to the body and, appropriately used, it can impact on physiological states as well as widen psychological experiences. For example, the psychophysiological study by Tang Poy and Woolhouse found that seeing others dance in synchrony impacts on aesthetic appreciation and causes pupil dilation, a measure frequently used for cognitive and emotional processing. Whereas, using electrodermal activity (EDA), Fontanesi and DeSouza also employed a psychophysiological measure for emotional arousal. The authors found that EDA was significantly higher during dancing than during an exercise class. Also after dancing, people with Parkinson's reported significantly higher self-efficacy (which includes a beauty subscale) compared to after exercise. The authors conclude that intrinsic artistic elements of dance may influence affective responses which have been evident in self-report and direct measures taken from the body.

Strong evidence for psychological improvements come from the multi-centred randomized controlled trial with over 100 participants who suffer from depression in Finland (Hyvönen et al.; Pylvanainen et al.). This work gives us confidence that in the hands of qualified dance movement therapists, the positive impact of dance can be substantial for people who struggle with depression on both reducing depression scores as well as improving body image. These positive results can be sustained over at least 3 months after the completion of the intervention. Smaller pilot randomized controlled trials conducted in Latvia and the UK indicate similar positive outcomes. The Latvian study (Majore-Dusele et al.) for example, showed that women with chronic pain who attended dance movement therapy groups not only decreased levels of depression compared to the treatment as usual group but also lowered their pain intensity. The study on autism in the UK (Aithal et al.) indicated that both social communication as well as overall wellbeing were improved in the dance movement therapy groups for all children, including both those with verbal as well as those with non-verbal skills.

It is notable that in the papers included in this Research Topic, methodological designs that follow a health-related hierarchy of evidence are adopted such as randomized controlled trials (Hyvönen et al.; Majore-Dusele et al.; Aithal et al.) or systematic reviews as is the case with the study from the Netherlands on personality disorders (Kleinlooh et al.). Most studies however, involved a mixture of hard and soft data with only a few studies relying exclusively on qualitative information (Sundstrom and Jola; Kipling-Brown and Gray; Payne and Brook); interviews on user perspectives, autoethnographic approaches and action research were some of the approaches adopted in the qualitative studies included in this compilation of papers. In a complex art form such as dance, attending to both what is nuanced and internal as well as what is measurable and external appears to be the way researchers approach this subject.

Overall, it seems that dancing makes us joyful, happy, and connected across generations and despite illnesses with authors exploring why dance is so good for us in psychological as well as physiological terms. Similar explorations can be found in the studies relating to theater, drama therapy and psychodrama.

## Theater, Drama Therapy, and Psychodrama

Ten studies in this Research Topic focused on how theater and drama based processes promote social and psychological health, and address specific concerns across the lifespan, with half of these focused on adults.

In one of the two studies on theater, Panero and Winner's quantitative study in the U.S. suggested acting students scored higher than non-acting students did on some components of flow and of empathy in their everyday lives, which could indicate positive psychological experiences of acting. Actors' dissociation rose significantly post-performance, however, which could indicate negative psychological experiences of acting. None of these characteristics were related to acting quality as judged by independent raters. García-Arch et al. conducted a quantitative controlled study in Spain showing that a 14-day performing arts program designed to shift biased attitudes toward physical illness among the general population could be effective in reducing levels of implicit bias toward illness.

In drama therapy, a 10-week manualized protocol was used to enhance maternal insightfulness and reduce children's behavior problems in mixed-method study (Feniger-Schaal and Koren-Karie). The findings indicate improved maternal insightfulness and a corresponding decrease in children's externalized and general behavioral problems. In another drama therapy study, a qualitative grounded theory approach was used to explore the benefits of a 16-week group drama therapy protocol with adolescents with an identified intellectual/developmental disability (Musicka-Williams). The findings highlight the value of dramatic imitation not only as an entry point to the dramatic work but also in facilitating relational connection to others in the group. A different mixed methods study examined the benefits of a 10-month drama therapy group culminating in autobiographical therapeutic performance with ten court involved adults (Ray and Pendzik). The pre- and post-interviews and self-report measures indicated that participants experienced a sense of self-acceptance and understanding, empathy for others, and a potentially improved ability to engage in human service programs designed to increase job readiness and enhance adaptive living skills.

In a psychodrama study, Kushnir and Orkibi illuminated four benefits of concretization as a mechanism of change: reducing the ambiguity of the problem, externalizing the protagonist's problem, enhancing the therapist-protagonist therapeutic bond, and bypassing the protagonist's defense mechanisms, based on interviews with seasoned psychodramatists. The operationalization of concretization that is offered could be useful for future experimental examination. In a different qualitative study from Israel, an open psychodrama group with abused women residing in domestic violence shelters was observed to reduce anxiety, stress, guilt and self-blame while reinforcing perceptions of self-worth and confidence (Ron and Yanai). Giacomucci and Marquit's quantitative single group pretest-posttest study explored the effectiveness of trauma-focused group psychodrama in the treatment of post-traumatic stress disorder (PTSD) at an inpatient addiction treatment center in the U.S. The results suggest clinically significant reduction in overall PTSD symptoms, but this should be taken with caution given the lack of control group and clients' exposure to other treatments while treated in psychodrama. In a mixed methods psychodrama study in Portugal, Pires et al. examined the therapeutic use of masks created by five adolescents suffering from anxiety. The most significant experiences quantitatively reported by participants during sessions were awareness, insight, self-understanding, empowerment, and relief. At the end of the study, participants also qualitatively reported a perceived increase of calmness and world connection, satisfaction in interpersonal communication, and better emotional expression and regulation. Importantly, the mask technique was experienced as playful and engaging. In Italy, Testoni et al. explored the effects of a death education project on 89 high school students coping with traumatic grief due to the death of two classmates. The intervention involved eight 2-h meetings of psychodrama aimed at helping students elaborate their feelings, as well as meditation. A battery of quantitative self-report measures indicated that the intervention reduced students' fear of death and its avoidance, normalized death as something natural, and improved wellbeing.

On the whole, these studies demonstrate that theater based processes can increase our empathy, reduce bias, deepen insight and self-acceptance, strengthen relationships, and impart both a sense of relief and vitality. Managing psychological distress as well as developing socially-needed skills were some important highlights from this group of studies.

## Music and Music Therapy

Fifteen studies in this Research Topic focused on the benefits of music listening, music making, and music therapy. Five studies focused solely on music, 10 studies of music therapy, and covered populations across the lifespan. Studies were carried out with people with intellectual disabilities, dementia, depression and people living with and beyond cancer. The remaining studies were conducted with non-clinical samples. Quantitative, qualitative and mixed-methods studies examined the impact of music listening and music making. Further, two studies examined neurophysiological responses to music. The benefits of music and music therapy included improvements in wellbeing, quality of life, mood and emotion, anxiety, stress and distress and growth mindset. A range of changes in social functioning and social processes were also noted. For instance, increased communication, confidence and social interaction, building resilience and political agency and supporting relationships.

Some music therapy studies that are worth drawing the reader's attention to include the randomized controlled trial by Elkkila et al. in Finland, the study by Clark et al. in Australia and by Chong and Yun in South Korea. Erkkilä et al.'s randomized controlled trial examined the efficacy of a music therapy approach to treating adults with depression. Effects were large and significant on depression when improvisational music therapy was combined with the breathing exercise and listening homework. In Australia, Clark et al. also found a songwriting program was not only feasible and acceptable in people with dementia and their caregivers; there was also a large reduction in depression and improvements in mood next to improved cognitive performance and social connection. Chong and Yun adopted a mixed methods design to study the impact of music therapy on young offenders. The resource-oriented program led to improvements in self-concept, stress coping skills, and resilience.

Studies that focus on the content of music and music therapy interventions are also included here. Scrine for example, critiques the dominant trauma paradigms. Instead, she highlights how music therapy can challenge power relations by enabling young people to reframe perceived risk, foster resistance and building political agency, while encouraging young people to challenge assumptions of safe spaces and instead move toward practices of structuring safety. de Witte et al. analyzed focus group interviews with music therapists who worked with people with mild intellectual disabilities where they focused on stress reduction. The analysis revealed that active interventions led to synchronizing, tension release, and direct relaxation. Tempo and the dynamics of the music were the most critical to consider when selecting music interventions for stress reduction.

There are also some interesting studies in community music and music education included in this collection of studies that highlighted the value of music for emotional and social skill development, growth and wellbeing. A mixed-methods analysis of a community music programme for people with disabilities identified improvements in self-expression, confidence, mood, and social skills. There were also increases in communication, social interaction and joint interaction. Music-making was recognized as an important non-verbal mode of self-expression (MacGlone et al.). Attention to growth and attainment is given by Holochwost et al. This team found that children at risk of poverty who were enrolled in orchestral music education programmes had significantly higher levels of growth mindset, with evidence of a dose-response relationship for 2–3 years of participation in such programmes. A new measure of eudaimonic music listening functions was developed by Groarke and Hogan which measures peak experiences, flow and transcendence in music listening experiences. Scores were associated with higher positive and negative affect, indicating that eudaimonic music listening is associated with greater emotional experience, rather than greater emotion regulation. A qualitative interview study by Krause and Davidson found that among older Australian adults in residential care music listening supported their wellbeing by providing entertainment, enjoyment, relaxation and mood regulation.

There is also evidence that music and music therapy can be of value for people living with cancer. For example, the systematic review and meta-analysis by Kohler et al. found that music therapy significantly improved psychological wellbeing, quality of life and significantly reduced physical symptom distress among adults with cancer. A pilot study involving music-based play activities aimed at managing distress for children undergoing hematopoietic stem cell transplantation (HCST) and their caregivers was found to be feasible and acceptable (Holochwost et al.).

Hunt et al. also focused on cancer care, but they used EEG in a mixed-methods design with case series to examine the neuronal effects of listening to entrainment music compared with preferred music in patients with chronic cancer pain. Analysis showed common brain responses to personalized entrainment-healing music in theta and low beta range in right pre- and post-central gyrus. The authors observed somatosensory changes consistent with processing pain during entrainment-healing music that were not seen during preferred music. Bower et al.'s systematic review which synthesized brain imaging data about the neural processing of music in children, found infants and children process music more slowly and utilize different cortical areas compared to adults.

Both of these two studies are important for reporting on the impact of music on the brain and begin to build a picture of how not only music, but maybe also all the arts can have a major positive physiological impact. Looking at the impact of all the arts in different combinations have also been included in this call, as we see in the section following.

## Studies Combining Artistic Disciplines and Multiple Arts Therapies

Six studies examined the impact of interventions that involve multiple arts forms in educational and combined arts therapies in clinical contexts with people across the lifespan. Two randomized controlled trials are worth flagging up here: the study by Moula et al. in the UK and the study by Ho et al. in Hong Kong. In a pilot randomized controlled trial, Moula et al. investigated the effectiveness of arts therapies interventions (music, drama, dance movement and art therapy) on child- and teacher-reported mental and physical outcomes. In a sample of 62 children, the results indicated not only significant improvements in life functioning and improvement of overall wellbeing, but also significant improvements in childrens' duration and thus, quality of sleep, making a strong case that the arts therapies can have a positive impact on physical and emotional health of children. The trial conducted by Ho et al. in Hong Kong involved over 100 Chinese adults with intellectual disabilities, who were randomized into an expressive arts-based intervention group and a control group of routine care. Although, overall, the quantitative results indicated no intervention effects on wellbeing, behavior, and mood, analysis of drawings pre-post intervention indicated that those in the intervention group used a wider variety of colors which may imply a more positive state of emotional wellbeing. Also, the interview findings suggested that those in the intervention group were more emotionally expressive and stable after the intervention.

In all cases, the need for further research is evident, research that can benefit from work across disciplines. For example, the effects of school-based arts education on various competency outcomes were examined in a systematic review by Schneider and Rohmann. The authors concluded that the evidence across arts domains, and for different outcomes, remains limited due to small sample sizes, small number of studies, and a range of effect sizes. In a complementary conceptual article, Holochwost et al. provides guidance of how the quality of the studies can be improved, arguing that “arts education” researchers should (1) define the specific domain of the arts education activity (e.g., acting vs. drawing vs. singing), (2) describe the contexts in which the activity occurs (e.g., handled by a facilitator vs. taught by a professional artist), (3) explain the socioemotional development concept targeted in the study (e.g., self-concept in adolescence), and (4) take into consideration participants' demographic information (e.g., socioeconomic status, gender, and ethnic/racial identity).

The importance of laying out clearly what is responsible for change has been an important motivation for the study by de Witte et al. who conducted the first scoping review on arts therapies therapeutic factors. For further randomized controlled trials to take place, it is crucial that researchers are clear of what are the active ingredients of interventions. Through the 67 studies included in this review, the authors distinguish between factors specific to a particular arts therapies discipline, factors shared by all arts therapies disciplines, and factors that are common across all psychotherapies. Also, 19 domains of therapeutic factors were identified, of which three domains (“embodiment,” “concretization,” and “symbolism and metaphors”) were composed solely of factors unique to the arts therapies. The authors offer a detailed evaluation of the findings, suggestions for future research, and discuss implications for clinical practice and training.

Finally, Vulcan conducted a phenomenological-hermeneutic qualitative study that explored the particular challenges faced by 28 therapists in Israel who work with children who have an autism spectrum disorder. The therapists were from diverse orientations: dance/movement, arts, music, and drama therapists, clinical psychologists, and clinical social workers. The findings underscore the key role of the concrete body in intersubjective relationships and in the therapeutic process, pointing to the potential impact of somatic and kinetic interventions.

## Summary

The 82 articles included in this Research Topic affirm the value of the arts as a cost-effective global resource for keeping us well, living fuller lives and meeting major challenges facing health and social care such as aging, implicit bias, chronic medical conditions, and mental health. And as promised, they also offer valuable evidence and insight into the psychological, physiological, and also the social benefits of the arts. Each contribution advances our understanding of what works where and with whom and offers methodological roadmaps and recommendations for future research. Collectively, the papers in this compilation also offer opportunities for cross pollination highlighting the benefits from working across a continuum of care from community arts, arts and health and arts therapies.

Over 20 studies look at the benefits of the arts and arts therapies for children and adolescents in mainstream education, special schools or hospitals. The remaining studies look primarily at adult populations ranging from members of the general public to vulnerable groups and individuals with medical conditions. Older age groups also receive substantial attention with Parkinson's and dementia being two areas repeatedly reported on.

Several studies in this call argue that the arts can act as social glue that brings together families, generations, and wider communities. For example, the intergenerational and community interventions using dance by Douse et al., and Shuper-Engelhard show the power of dance to bring people together. Participatory music has been researched for its capacity to enable expression (MacGlone et al.), growth and attainment (Holochwost et al.), wellbeing (Groarke and Hogan), while may offer entertainment, enjoyment and relaxation (Krause and Davidson) for children, adults and older people alike. Findings from García-Arch et al. suggest that a performing arts intervention program reduced levels of implicit biases among the general public. Acting training, in particular, was related to higher levels of emotional, cognitive, and compassionate empathy in everyday life (Panero and Winner). Several studies focused on short-term and long-term interventions of visual arts and art therapy improving socio-emotional skills in adolescents (Kastner et al.) as well as the expression (Harpazi et al.) and regulation of emotions (Drake) in children. Other studies examined how art could improve quality of life and wellbeing in clinical (Savazzi et al.; Tong et al.) and non-clinical adult populations (Pipere et al.).

We have some large multi-centered randomized controlled trials that argue convincingly, through robust designs, that there is a significant power in dance movement and music therapy to lower depression (Hyvönen et al.; Erkkilä et al.). Similar impact on reducing anxiety and depression is reported in art therapy (Havsteen-Franklin et al.), while evidence is presented on the contribution of psychodrama to reducing symptoms of trauma (Yanai and Ron; Giacomucci and Marquit), adding to the substantial growth of evidence of the effectiveness of the arts therapies in reducing symptoms of ill health. In order to further advance our understanding of direct cause-and-effect and generate strong empirical evidence, it is necessary to engage further with randomized controlled trials that look at the effectiveness of the arts with different populations as well as experimental studies that target a smaller number of specific quantifiable components enriched by an in-depth understanding of subjective experiences. We feel that with financial support, the arts and arts therapies can be implemented in a powerful, and more targeted manner.

Contemporary developments in the use of digital technologies are reported in the art therapy publications (Zubala et al.; Shamri Zeevi; Kaimal et al.), responding not only to the needs created by the pandemic but also to the longer term and evolving artistic digital language spoken by the diverse populations we serve. It is expected that as digital technologies are growing in sophistication, research in the other artistic practices will also grow, supporting embodied 3D real time artistic experiences with important psychological, physiological and/or social value.

Finally, there are studies that highlight the impact of the arts and arts therapies on neurophysiology, motor skills, physical health and other biomarkers such as studies by Bower et al. and Hunt et al. on the impact of music on the brain, by Moula et al. on the impact of arts therapies on the quality of sleep of children and the physiological and neurological benefits of engaging in visual arts and art therapy for Alzheimer's patients (Savazzi et al.). The use of wearables, EEG, FMRI scans and similar devices are gaining popularity, attempting to capture ways in which our physiology is directly affected by not only watching the arts but also by active participation. The interest from the neuroscientific community in the arts is clearly evident. However, expensive equipment also requires equivalent financial investment. Similar investment is needed for large, multi-centered randomized controlled trials. Research in the arts have been traditionally under-resourced; for large and expensive studies to take place that can fully de-code why the arts are so impactful for our mental health, a similarly large amount of financial investment is required.

For now, it is possible that some of the therapeutic factors proposed by the systematic review by de Witte et al. for the arts therapies may also hold true for several different uses of the arts. It is likely for example, that the potential of the arts to support full immersion in arts-making processes (“embodiment”), to make tangible what may be difficult to articulate or comprehend (“concretization”) and to simultaneously clarify experience while maintaining complexity and possibility (through “symbolism and metaphors”) can be an answer to a lot of our struggles and difficulties.

While research on the mechanisms that are responsible for change through the arts is ongoing, so are studies on the outcomes from engagement in the arts. The range of benefits of the arts and arts therapies that are included in this rich compilation of studies is impressive, the results are promising and the future research possibilities proposed are exciting. Our editorial team hope that this Research Topic is not only enjoyable, but also highly stimulating, offering opportunities for future growth in this exciting area of research and practice.

## Author Contributions

JD and JC-C: wrote the art and art therapy section. CJ and VK: wrote the dance and dance movement therapy sections. NS, HO, and MEP: wrote the drama/theater and drama therapy/psychodrama section. JG and FB: wrote the music and music therapy section. HO, MEP, and NS: wrote the mixed arts and mixed arts therapies section. VK and NS: wrote the introductory and concluding sections of the editorial. VK, NS, and HO: edited and proof-read the whole piece. All authors contributed to the editorial focusing on their own specialty.

## Conflict of Interest

The authors declare that the research was conducted in the absence of any commercial or financial relationships that could be construed as a potential conflict of interest.

## Publisher's Note

All claims expressed in this article are solely those of the authors and do not necessarily represent those of their affiliated organizations, or those of the publisher, the editors and the reviewers. Any product that may be evaluated in this article, or claim that may be made by its manufacturer, is not guaranteed or endorsed by the publisher.
